# The effects of training female students in emotion regulation techniques on their social problem-solving skills and social anxiety: a randomized controlled trial

**DOI:** 10.1186/s13034-025-00860-1

**Published:** 2025-01-22

**Authors:** Ashraf Akbari, Camellia Torabizadeh, Narjes Nick, Giti Setoodeh, Parvin Ghaemmaghami

**Affiliations:** 1 Student Research Committee, School of Nursing and Midwifery, Shiraz University of Medical Sciences, Shiraz, Iran; 2 Community Based Psychiatric Care Research Center, Shiraz University of Medical Sciences, Zand St., Namazi Sq., Shiraz, Iran; 3 Department of Community Health Nursing, School of Nursing and Midwifery, Shiraz University of Medical Sciences, Shiraz, Iran; 4 Department of Mental Health and Psychiatric Nursing, School of Nursing and Midwifery, Shiraz University of Medical Sciences, Shiraz, Iran; 5 Biostatistics, School of Nursing and Midwifery, Shiraz University of Medical Sciences, Shiraz, Iran

**Keywords:** Emotion regulation, Social anxiety, Problem solving, Group training, Students

## Abstract

**Background:**

Social anxiety is one of the most prevalent anxiety-related disorders among adolescents with many adverse effects on the social and academic lives of this population. In addition, poor social problem-solving skills can aggravate anxiety in individuals suffering from anxiety. Emotion regulation can help adolescents control and moderate their feelings, thereby enabling them to understand their emotions better, cope with their negative emotions in a positive way, and adopt a more realistic approach to solving their problems. The objective of the present study is to investigate the effects of educating female students in emotion regulation techniques on their social problem-solving skills and social anxiety.

**Method:**

This study was a randomized controlled trial conducted in Iran, utilizing a pretest-posttest design with control and intervention groups. The subjects were 47 female high-school second graders who were randomly divided into a control (25 students) and an intervention group (22 students). The intervention group was collectively educated in emotion regulation techniques in weekly one-hour sessions for eight weeks. The control group did not receive any intervention. The social anxiety and social problem-solving scales were completed by both groups before, immediately after, and one month after the intervention. The collected data were analyzed in SPSS v. 22 and level of significance was set at *p* < 0.05.

**Result:**

Data analysis of the intervention group compared to the control group demonstrated that group training in emotional regulation techniques effectively increased social problem-solving skills scores (*p* = 0.003) and decreased social anxiety scores (*p* < 0.0001) among students in the intervention group compared to their pretest scores. These effects remained stable during the follow-up phase.

**Conclusion:**

In view of the prevalence of social anxiety among adolescents, it is suggested that the policymakers and administrators in the education system promote emotion regulation skills in adolescent students to facilitate their psychological adaptation and improve their emotional capabilities.

**Trial registration:**

The present study was registered under the code IRCT20220413054521N1 (Registration date: 27/02/2024) in the Iranian Registry of Clinical Trials.

## Background

Adolescence is the most sensitive and critical stage of growth in one’s life. At this time, individuals search for their identity and autonomy and, because of instabilities in their psychological state, encounter a variety of challenges which can lead to behavioral and mental disorders, including anxiety [1–3]. In 2019, anxiety disorder ranked eight on the Global Burden of Mental Illness; among individuals aged 15 to 24 years, anxiety is the fourth leading cause of Years Lived with Disability [4]. A 2019 report by the American Academy of Pediatrics showed that one in three of adolescents aged between 13 and 18 suffered from anxiety disorder [5]. Also, in 2016, the National Survey of Children’s Health found that 7.1% of children aged between 3 and 17 years had anxiety-related issues [6].

One of the most serious types of anxiety disorders among adolescents is the social anxiety disorder [7], which is characterized by great manifest fear of negative evaluation by others in social interactions and situations, e.g. meeting strangers, making a speech, and eating and drinking in front of others [8]. This fear can preclude students’ active participation in social situations and communicating with others [7]. The rate of social anxiety among children and adolescents in Australia and Spain is 2.3% and 4% respectively [9, 10]. The yearly prevalence of this disorder in the U.S. has been estimated at 7% [8]. A study in Sweden reported that the yearly prevalence of the social anxiety disorder among the adolescents in this country was 16% [11]. In Iran, the rate of social anxiety among children and adolescents nationwide was reported to be 2% [12], while a study conducted in one province found the rate of social anxiety among high-school students to be 22.30% [13]. Females are more prone to social anxiety and this gender difference is more noticeable among adolescents [8]. The symptoms of the social anxiety disorder have significant adverse effects on the education and employment of adolescents, leading to disruptions in the physical, mental, social, and emotional aspects of their quality of life [14].

One of the crucial skills in improving one’s mental health is the social problem solving-skills [15]. Problem solving is a cognitive-behavioral process which enables individuals to find effective strategies for coping with their daily problems [16]. Individuals who are skilled in problem solving have greater self-confidence and are willing to face their problems and find a solution to them. When problems come up, these individuals are able to manage their stress and experience less anxiety [17]. In addition, when individuals are involved in problem solving, the emotional, cognitive, and behavioral aspects of their personality interact dynamically, which facilitates or controls the process [18]. Proper management and regulation of emotions contribute to satisfactory problem-solving outcomes. Thus, an effective factor in problem solving is emotion regulation [19, 20]. Emotion regulation incorporates all the conscious and unconscious strategies which are applied to strengthen, maintain, or moderate the emotional, behavioral, and cognitive components of an emotional reaction [21].

Education in emotion regulation is a psychotherapy-based treatment for anxiety disorders [22] and has been assessed and found to be effective in several studies [23–25]. A variety of approaches to learning emotion regulation have been suggested [21, 26], among them the emotion regulation techniques introduced by Leahy et al. [27], whose effectiveness has been confirmed in several studies [28, 29]. The purpose of these techniques is to regulate and moderate emotions and prevent negative and destructive reactions [30]. Educating adolescents in emotion regulation enables them to have a better perception of their feelings and emotions, distinguish between logical and illogical thoughts, and face their problems realistically [28]. However, their learning is influenced by the manner in which these techniques are presented and taught. One of the useful learning methods in health promotion is group training and intervention [31]. In group training, learners are divided into smaller groups, allowing for effective communication between the members of the groups and better interaction with the educator, which results in better learning [32].

The prevalence of social anxiety among Iranian adolescents is lower compared to the aforementioned countries. However, studies conducted in Iran indicate that female adolescents with social anxiety face psychological challenges, such as low self-confidence, lack of self-acceptance, and low life expectancy [33, 34], which, if not properly addressed, will negatively impact the personal and social dimensions of the adolescents’ future lives. Therefore, it is important to design and implement interventions to address this disorder among Iranian female adolescents. Despite their adverse effects, psychological disorders are largely neglected in developing countries and research in the field of psychological pathology remains scarce in these countries [14, 35]. Since the symptoms of social anxiety are more common among adolescents, whose personal and social lives are adversely affected by them, schools should identify vulnerable adolescents and provide them a with a choice of interventions according to their needs, so that they can be guided to paths of growth [14, 36]. The effectiveness of models of emotion regulation training and the relationship between emotion regulation and symptoms of anxiety and depression have been investigated in several studies [23–25, 37]. However, an extensive review of literature showed that the effects of emotion regulation training on female students’ social problem-solving skills and social anxiety had not been addressed. Accordingly, the present study was conducted to measure the effects of educating female students in emotion regulation techniques on their social problem-solving skills and social anxiety.

## Methods

### Study design and participants

This was a pretest-posttest randomized controlled trial study with two groups, a control group and an intervention group, and a one-month follow-up period. The target population comprised female students from grades 10 to 12. The sample size was calculated based on relevant research [38] and using G*Power3 software. Using this software, with α = 0.05, β = 0.10, 95% confidence interval, 90% power, effect size (Cohen’s effect size of 0.845 and f = 0.42), correlation of 0.3 between measurement times, and considering a minimum 20% attrition rate, the final sample size was determined to be 50 participants (25 in the control group and 25 in the intervention group).

To collect data, first the researchers randomly selected one of the four education districts in the city where the study was conducted. Next, two public high-schools (excluding leading schools and schools for gifted students) were randomly selected from that district. After making arrangements with the school authorities, the researchers distributed the demographics survey among the students. From each school, based on the inclusion criteria and using a random number table, 25 students were selected and assigned to one of the control or intervention group. Inclusion criteria: (1) being a female secondary high-school student, (2) being willing to participate in the study, (3) written informed consent from each student and her parents, (4) not having received psychological interventions during or at least six months before the study, (5) living with both parents at the same time, and (6) not having participated in a similar study or training program before.

Exclusion criteria: (1) having a known psychological illness of any kind, (2) having a physical illness which could have affected their performance (regularly participating in the training sessions, participating in the group discussions, and doing assignments), (3) having experienced a crisis, e.g. losing a close family member (a grandparent or a sibling) in the past six months, (4) having been transferred from another town, and (5) missing more than a session of the intervention.

### Procedure

**Fig. 1:**
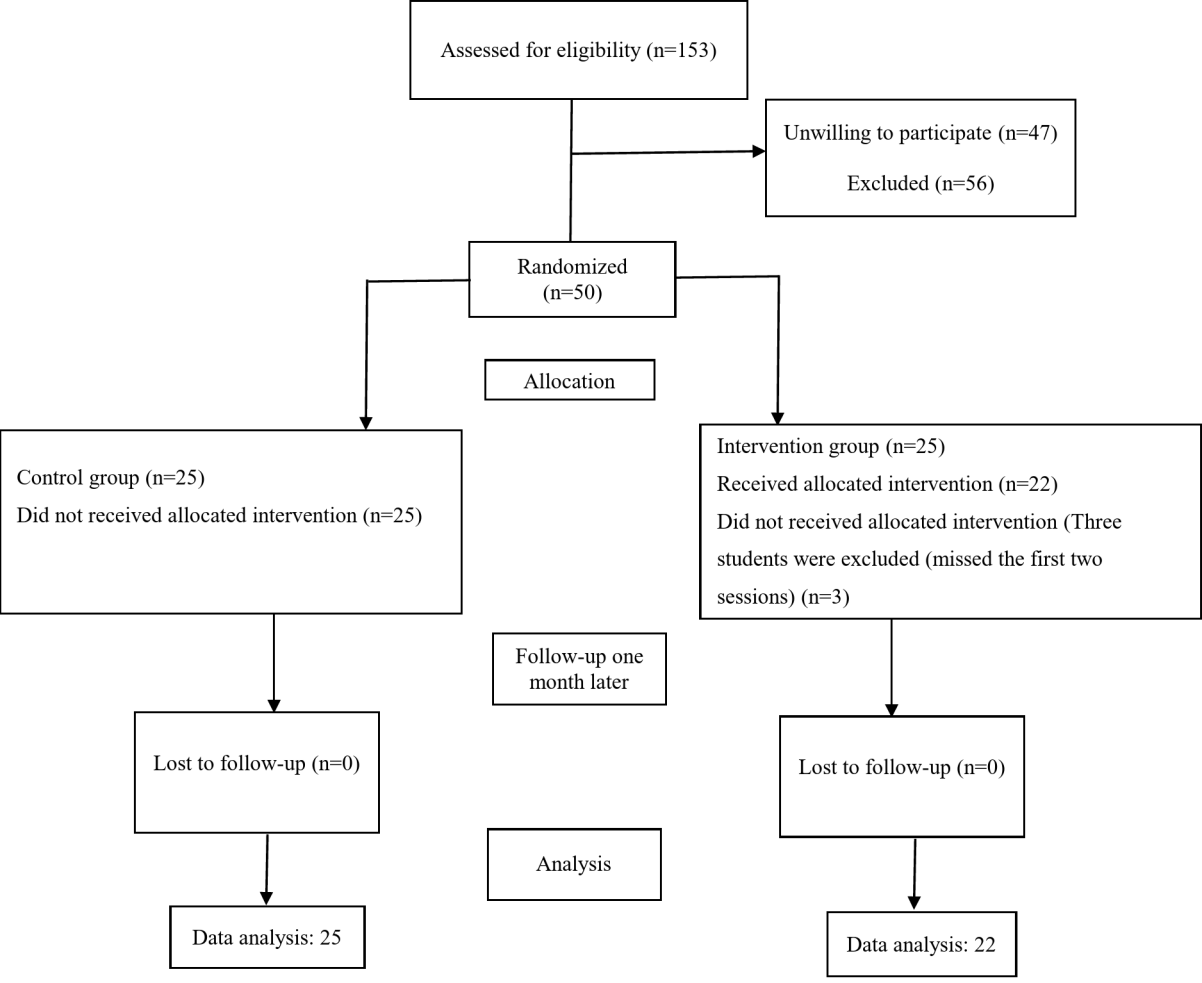
CONSORT flowchart of the study

The demographics survey was completed by 153 students from two schools in the same educational district. Fifty-six of these students did not meet the inclusion criteria, and of the remaining 97 students, 47 were not willing to participate in the study. The remaining 50 students and their parents, who were willing to participate in the study, gave their written informed consent. The students were randomly divided into a control (25 students) and an intervention group (22 students). Three students in the intervention group missed the first two sessions of the training and were thus excluded. Thus, 22 students from the intervention group were trained and went through the pretest, posttest, and follow-up (one month later) stages. As for the control group, none of the participants was excluded and all the 25 went through the pretest, posttest, and follow-up (one month later) stages. Figure 1 shows the CONSORT flowchart of the study.

### Intervention group

The education in emotion regulation techniques was carried out in weekly group sessions, each lasting one hour, for eight consecutive weeks. The sessions were held in the school classrooms. The content of the education was created according to the set of emotion regulation techniques developed by Leahy et al. [27] and approved by the professors at the faculty of psychiatric nursing. The intervention was executed by the first researcher and supervised by the psychological health expert on the research team. At the end of each session, assignments related to the educational content of that session were given to the participants. In the following session, the assignments were checked and the materials from the previous session were reviewed. In the first session, the participants were informed about the objectives and stages of the study, the meaning and significance of emotion regulation, and different types of positive and negative emotions. From session two through session seven, the participants were trained in one emotion regulation technique every session. In session eight, all the presented materials were recapped. Table 1 shows a summary of the content of the sessions.

**Table 1:** A summary of the content of the emotion regulation training sessions

Session	Beginning	Educational content	Ending
1	1. The researcher introduced herself. 2. The participants were introduced to each other. 3. The primary and secondary objectives, logic, and framework of the study were explained. 4. The pretest was given.	Definition of emotion regulation; the significance of emotion regulation; positive and negative emotions	1. Recap 2. Assignments
2	1. Checking assignments 2. Reviewing the previous session	The emotional labeling technique and distinguishing thoughts from feelings	1. Recap 2. Assignments
3	1. Checking assignments 2. Reviewing the previous session	The emotional normalization technique	1. Recap 2. Assignments
4	1. Checking assignments 2. Reviewing the previous session	The emotional acceptance enhancement technique	1. Recap 2. Assignments
5	1. Checking assignments 2. Reviewing the previous session	The emotional visualization technique	1. Recap 2. Assignments
6	1. Checking assignments 2. Reviewing the previous session	Enhancing emotional awareness technique	1. Recap 2. Assignments
7	1. Checking assignments 2. Reviewing the previous session	Weighing costs and benefits technique	1. Recap 2. Assignments
8	Checking assignments	Reviewing the presented techniques and final recap	The posttest was given.

### Control group

The control group did not receive any intervention in the duration of the study. The participants in this group were assessed before, immediately after, and one month after the intervention. In compliance with ethical considerations, at the end of the study, the content of the emotion regulation training sessions was provided to the control group in the form of a pamphlet.

## Measures

1. The demographics survey: Consisting of eight items, the survey addressed the students’ personal and social characteristics: age, grade, whether or not the student has been transferred from another school, residence, having experienced a crisis in the past six months, suffering from any physical or psychological diseases, having received a psychotherapeutic intervention (medication or counseling) in the past six months, and which of her parents the student was currently living with.
2. LaGreca Social Anxiety Scale for Adolescents: Developed and tested by LaGreca (1999), this scale consists of 16 items and three subscales: fear of negative evaluation, social avoidance and distress in new situations, and social avoidance and distress in general situations [39]. The items are scored on a 5-point Likert scale, and the score range is between 16 and 80 [40]. The three-factor structure of the original version of the Social Anxiety Scale for Adolescents has been validated in several studies [41–44]. The reliability of the original version of the scale measured by the test-retest method has been reported to range from 0.54 to 0.75 for an eight-week period [43] and from 0.47 to 0.75 for a six-month period [44]. The psychometric properties of the Farsi version of the scale were measured in Iran by Ostvar and Razavie, who verified the three-factor structure of the scale after it was completed by a sample of Iranian adolescents. The test-retest reliability of the social anxiety scale was measured using a sample of 50 students with a three-week interval and found to be 0.88. The internal consistency of the subscales of fear of negative evaluation, social avoidance and distress in new situations, and social avoidance and distress in general situations were reported to equal Cronbach’s alphas of 0.84, 0.76, and 0.77 respectively [40].
3. The Social Problem-Solving Inventory-Revised: Developed and tested by D’Zurilla et al. (2002), this scale consists of 25 items and five subscales: positive problem orientation, negative problem orientation, rational problem-solving style, avoidance problem-solving style, and impulsivity/carelessness problem-solving style [45]. The items are scored on a 5-point Likert scale, and the score range is between 25 and 125 [46]. The test-retest reliability and Cronbach’s alpha of the original version of the inventory were reported to be between 0.68 and 0.91 and 0.69 and 0.95 respectively [47]. The psychometric properties of the Farsi version of the inventory were measured in Iran by Mokhberi et al. To determine the validity of the inventory, the researchers measured the correlation between the factors of the instrument and found them to be between 0.32 and 0.85, which were significant. The mean of the test-retest reliability coefficients of the instruments was 0.71, and its total Cronbach’s alpha was reported to be 0.85 [46].

### Outcome assessment

The La Greca Social Anxiety Scale for Adolescents and the Social Problem-Solving Inventory-Revised were used to evaluate both groups during Pretest, post-test, and follow-up. These scales were completed by the participating students on a self-report basis via paper questionnaires.

The intervention group participated in eight sessions of group training in emotion regulation. The control group did not receive any intervention. One of the researchers explained the study objectives and methodology to the students. Additionally, a written consent form was provided to the students and their parents, and the students and their parents were requested to complete the written informed consent form if they were willing to participate in the study.

### Data analysis

In the present study, the quantitative variables were presented as means (M) and standard deviations (SD), and the qualitative variables were presented as frequencies (%). When being analyzed by repeated measures ANOVA based on time (pretest, posttest, and follow-up) and group (intervention and control), all the findings were compatible. The intergroup independent variables were training in emotion regulation techniques and the control group. The intragroup independent variables were the pretest, posttest, and follow-up. Social anxiety and social problem-solving skills were the dependent variables. The normality of the data and homogeneity of the variances were tested by the Kolmogorov–Smirnov test and Levene’s test respectively. The collected data were analyzed in IBM SPSS Statistics v. 22. The level of significance was set at *p* < 0.05.

## Results

### Sample characteristics

The means of the ages of the students in the intervention and control groups were 16.89 ± 0.93 and 17 ± 1.31 years respectively. As for the students’ grades, 17 were in the tenth grade, 10 were in the eleventh grade, and 20 were in the twelfth grade. Three of the students in the control group and one in the intervention group had a physical disease, but since their illness did not affect their abilities and performance, they were not excluded. For analysis of the variable of age between the two groups, because of lack of normality, the non-parametric Mann-Whitney was used and the results showed that there was not a statistically significant difference between the two groups’ means (*p* > 0.05). To investigate the correlation between the qualitative variables of grade and having a physical or psychological disorder, the researchers used the Chi-square test and Fisher’s exact test. The results of these tests showed that, in terms of the variables in question, the differences between the two groups were not statistically significant (*p* > 0.05). In other words, the students in the two groups were homogeneous with regard to the above-mentioned variables. A summary of the demographic characteristics of the students is given in Table 2.

**Table 2:** Frequency distribution of the qualitative demographic variables per group

Variable	Control group (*n* = 25) M ± SD	Intervention group (*n* = 22) M ± SD	*p* value
Age	17 ± 1.31	16.89 ± 0.93	0.825
Grade			
Tenth grade, n (%)	8 (32)	9 (40.9)	0.096
Eleventh grade, n (%)	3 (12)	7 (31.8)	
Twelfth grade, n (%)	14(56)	6 (27.3)	
Having a physical or psychological illness			
Yes, n (%)	3 (12)	1 (4.5)	0.611
No, n (%)	22 (88)	21 (95.5)	

### Descriptive findings

The findings of the study showed a significant increase in the posttest and follow-up social problem-solving scores of the intervention group.

(*p* < 0.05). No change was noticed in the control group’s scores. The highest and lowest mean scores of both groups were for positive problem orientation and rational problem solving respectively. The intervention group’s posttest and follow-up social anxiety scores were significantly lower than the control group’s (*p* < 0.05). In the pretest stage, the highest and lowest mean scores of both groups were for the subscales of social avoidance and distress in general situations and fear of negative evaluation respectively (Table 3).

**Table 3:** The groups’ social problem-solving skills and social anxiety mean scores per time (before, immediately after, and one month after the intervention)

Variable	Group	Pre-test M ± SD	Post-test		*p*-value in within-group comparisons
			Immediately after intervention M ± SD	One month after intervention M ± SD	
Social Problem-solving skills	Intervention (*n* = 22)	76.40 ± 6.75	81.13 ± 6.49	81.18 ± 6.41	0.003
	Control (*n* = 25)	75.40 ± 7.13	74.96 ± 6.94	74.88 ± 6.38	0.883
*p-value* in between-group comparisons		0.622	0.003	0.002	–
Social anxiety	Intervention (*n* = 22)	52.54 ± 11.97	38.45 ± 11.1	33.22 ± 6.49	< 0.0001
	Control (*n* = 25)	44.80 ± 12.74	47.04 ± 12.77	48.60 ± 14.58	0.095
*p-value* in between-group comparisons		0.038	0.018	< 0.0001	–

### Inferential findings

The Kolmogorov–Smirnov test results showed that the distribution of the data for the pretest social problem-solving skills and social anxiety scores was normal (*p* > 0.05). The Levene’s test results confirmed that the error variances between the two groups were equal (*p* > 0.05). Also based on Mauchly’s test results (*p* < 0.05), the hypothesis of sphericity was true. Therefore, the researchers applied analysis of variance with repeated measures.

The results of analysis of variance with repeated measures showed that the intragroup variable of time had a significant impact on social problem-solving skills (f = 7.32, df = 1, *p* = 0.002) and social anxiety (f = 49.68, df = 1, *p* < 0.0001). In other words, regardless of the groups, there was a significant improvement in social problem-solving skills and social anxiety scores from the pretest stage to the posttest and follow-up stages and there was a statistically significant difference between the participants’ pretest, posttest and follow-up scores for these two variables (*p* < 0.05).

Intergroup analysis indicated significant changes in social problem-solving skills (f = 7.99, df = 1, *p* = 0.007): the intervention group’s posttest and follow-up scores were higher than the control group’s, which shows that training in emotion regulation techniques enhanced the intervention group’s social problem-solving skills. Intergroup analysis did not show a significant change in the variable of social anxiety (*p* = 0.095). Thus, for a better interpretation of the results, the researchers compared adjusted means using Bonferroni test.

Given that the comparison of mean social anxiety scores between the two groups showed statistically significant differences before the intervention, the impact of higher baseline social anxiety levels in the intervention group as compared to the control group was analyzed using repeated measures ANOVA. According to Table 4, the results of repeated measures adjusted by pretest indicate that the reduction in social anxiety scores in the intervention group was attributable to emotional regulation technique training, demonstrating the effectiveness of this training on the intervention group.

**Table 4:** Results of repeated measures adjusted analysis of emotional regulation technique training on students’ social anxiety

Source	Df	Mean Square	F	*p* value	Partial Eta Squared
Intercept	1	500.38	4.34	0.04	0.089
Pre-test	1	6079.77	52.76	< 0.0001	0.54
Group	1	6224.50	54.02	< 0.0001	0.55

Moreover, the interactive impact of group and time (stages of evaluation) was significant for the variables of social problem-solving skills (f = 4.63, df = 2, *p* = 0.012) and social anxiety (f = 42.38, df = 1.74, *p* < 0.0001). These findings demonstrate that the intervention group’s social problem-solving skills and social anxiety scores were significantly better than the control group’s in the posttest and follow-up stages (*p* < 0.05), verifying that training in emotion regulation techniques had a significant impact on the students’ social problem-solving skills and social anxiety. Analysis of the groups’ scores using Bonferroni test showed that the training resulted in better social problem-solving skills and social anxiety scores for the intervention group compared to the control group (*p* < 0.05). In addition, the participants’ follow-up scores for social problem-solving skills and social anxiety were not significantly different from their posttest scores, which proves that the impact of the intervention persisted (Table 5).

**Table 5:** The results of analysis of variance with repeated measures on the groups’ variables over three stages

Variable	Cause of change	Mean Square	Df	F	*p* value
Social Problem -solving skills	Impact of group	709.564	1	7.99	0.007
	Impact of time	165.470	2	7.32	0.002
	Interactive impact of group and time	106.742	2	4.63	0.012
Social anxiety	Impact of group	1025.311	1	2.91	0.095
	Impact of time	2196.591	2	49.68	< 0.0001
	Interactive impact of group and time	1896.892	1.74	42.38	< 0.0001

## Discussion

The present study was conducted to measure the effects of educating female students in emotion regulation techniques on their social problem-solving skills and social anxiety. Overall, group training in emotion regulation techniques improved the intervention group’s social problem-solving skills and lowered their social anxiety as compared to the control group.

The students who participated in the study had average social problem-solving skills. However, after being trained in emotion regulation techniques, the intervention group scored higher on the social problem-solving scale in the posttest and follow-up stages, and the impact of their training persisted through the one-month follow-up.

Similarly, the findings of a study in Belgium showed that training adolescents in adaptive emotion regulation skills centered around distraction, acceptance, cognitive reappraisal, and problem solving can effectively reduce the impact of sad feelings and increase the impact of happy feelings, eventually enhancing the social and emotional skills which are essential to overcoming the challenges in adolescence [48]. A study in the U.S. found that dialectical behavior therapy contributed to the emotional problem solving and social flexibility of the students in the intervention group [49]. In another study, gamification enhanced the self- regulation skills of students in solving math problems [50]. Benefiting from one’s emotion regulation skills when problems occur can help reduce negative emotional responses and lessen stress. These skills enable individuals to identify their automatic negative thoughts and manage them, which improves cognitive and emotional functions, including problem solving [51]. The findings of the abovementioned studies demonstrate that enhancement of emotion regulation skills and self-regulation in adolescents results in better management of social challenges and problem solving. Thus, training adolescents in emotion regulation techniques is an effective way to improve their social problem-solving skills.

In contrast to the findings of the present study, a study of emotion regulation and problem solving among gifted students reported that there was no significant correlation between emotion regulation techniques and problem-solving capabilities [52]. Likewise, another study of high-school students found a negative quantitative relationship between problem-solving and emotion regulation skills regarding problematic internet use [53]. These inconsistencies can be attributed to differences in study population and settings, gender and age of the participants, and the educational content and manner of educating the participants.

In the present study, the social anxiety level of the participants was found to be average. Training in emotion regulation techniques resulted in a significant decrease in the social anxiety of the intervention group, and the impact endured through the follow-up. On a similar note, the results of a study in Bangladesh showed that use of emotion regulation adaptive strategies correlated with a reduction in symptoms of anxiety as reported by adolescents [35]. Also, a U.S. study of the effects of emotion-focused cognitive-behavioral therapy on adolescents with anxiety disorders found that traditional cognitive-behavioral therapy and emotion-focused cognitive-behavioral therapy improved emotion management in both intervention groups. However, the participants in the emotion-focused cognitive-behavioral intervention group were able to manage their emotions better than before the intervention [25]. Another study in Turkey reported that a solution-focused group counseling program was effective in lowering anxiety in college students. The reduction in the students’ anxiety persisted through the three-month follow-up [54]. The results of a meta-analysis showed that emotion regulation training effectively reduced anxiety levels and should, therefore, be widely used [55]. These findings verify that learning emotion regulation techniques, e.g. emotional awareness and minimizing negative emotional responses to situations and replacing them with positive emotions, can decrease anxiety issues and facilitate adaptation with life events [56, 57].

Contrary to the findings of the present study and the abovementioned studies, a meta-analysis of the relationship between anxiety and cognitive regulation of emotion concluded that state anxiety did not correlate with emotion regulation adaptive strategies [58]. Also, a study of the relationship between emotion regulation strategies and anxiety in medical students found that the correlation between the two variables was not statistically significant [59]. These contradictory results can be attributed to differences in study population and settings, gender and age of the participants, and the educational content and manner of educating the participants in the studies.

Social anxiety disorders have a lasting impact on the personal relationships and professional and family lives of adolescents [60]. Yet, these disorders are frequently ignored and few adolescents with the symptoms of social anxiety seek treatment, but are willing to get help if health services are available [14]. In view of the findings of the present study, in addition to having their social anxiety levels measured, adolescents need to be trained in emotion regulation as an effective intervention in promoting their mental health.

### Limitations

In the present study, data were collected using questionnaires. It is possible that response bias inclined the participants to give inaccurate answers to the questions. However, by informing the participants about the objectives of the study and assuring them of confidentiality and anonymity, the researchers tried to control this confounding effect. The sample of the study was selected from the girls’ high-schools located in one town, which can limit the transferability of the findings to other towns or countries and male students. The absence of an active control group was another limitation of the study. In other words, factors, like the passage of time, could have had an effect on the participants’ scores. Future studies should examine the impact of emotional regulation technique training on social anxiety and social problem-solving skills in students with an active control group.

## Conclusion

The findings of the present study demonstrate that group training in emotion regulation techniques has a positive impact on the psychological skills of female high-school students. The intervention specifically improved the participants’ social problem-solving skills mean score and lowered their social anxiety mean score. Considering the prevalence of social anxiety among adolescents and the positive impact of emotion regulation training on management of feelings and coping strategies, it is suggested that policymakers and administrators in the education system promote emotion regulation skills in adolescent students to facilitate their psychological adaptation and promote their emotional capabilities.

## Data Availability

The datasets analyzed in the current study are available from the corresponding author on reasonable request.

## Abbreviations

ANOVA: Analysis of Variance
SPSS: Statistical Package for the Social Sciences
